# Morphological and physiological determinants of local adaptation to climate in Rocky Mountain butterflies

**DOI:** 10.1093/conphys/cow035

**Published:** 2016-09-22

**Authors:** Heidi J MacLean, Jessica K Higgins, Lauren B Buckley, Joel G Kingsolver

**Affiliations:** 1 Department of Biology, University of North Carolina at Chapel Hill, Chapel Hill, NC 27599, USA; 2 Department of Biology, University of Washington, Seattle, WA 98195, USA

**Keywords:** Climate change, *Colias*, flight

## Abstract

Flight is a central determinant of fitness in butterflies and other insects, but it is restricted to a limited range of body temperatures. To achieve these body temperatures, butterflies use a combination of morphological, behavioural and physiological mechanisms. Here, we used common garden (without direct solar radiation) and reciprocal transplant (full solar radiation) experiments in the field to determine the thermal sensitivity of flight initiation for two species of *Colias* butterflies along an elevation gradient in the southwestern Rocky Mountains. The mean body temperature for flight initiation in the field was lower (24–26°C) than indicated by previous studies (28–30°C) in these species. There were small but significant differences in thermal sensitivity of flight initiation between species; high-elevation *Colias meadii* initiated flight at a lower mean body temperature than lower-elevation *Colias eriphyle*. Morphological differences (in wing melanin and thoracic setae) drive body temperature differences between species and contributed strongly to differences in the time and probability of flight and air temperatures at flight initiation. Our results suggest that differences both in thermal sensitivity (15% contribution) and in morphology (85% contribution) contribute to the differences in flight initiation between the two species in the field. Understanding these differences, which influence flight performance and fitness, aids in forecasting responses to climate change.

## Teaser

We use field experiments to examine how butterflies achieve the flight required to forage, mate, and lay eggs in cool, montane environments. Potential adaptations include thermoregulatory traits that elevate body temperatures, basking behaviors, or shifts in the thermal sensitivity of flight. By comparing populations and species along an elevation gradient, we find that they use a combination of morphological, physiological, and behavioral adaptations to achieve flight.

## Introduction

Most ectotherms have a restricted range of body temperatures over which they can achieve high rates of resource acquisition, growth and other aspects of performance (Andrewartha and Birch, 1954; Magnuson *et al*., 1979; Huey and Hertz, 1984). Locomotion is a key aspect of performance in many ectotherms, and thermal constraints on locomotion can be important determinants of activity patterns, reproductive success and fitness (Adolph and Porter, 1993; Kearney *et al*., 2009a; Sinervo *et al*., 2010; Buckley and Kingsolver, 2012).

Terrestrial ectotherms can adapt evolutionarily to local climate conditions through shifts in behaviour or morphology that allow them to achieve preferred body temperatures or through physiological shifts in the thermal range of performance (Angilletta, 2009). Many insects adapt to local climates along elevation and latitudinal gradients through morphological differences in body size, coloration and insulation, allowing them to achieve higher body temperatures in cooler environmental conditions (reviewed by Mani, 1968; Hodkinson, 2005). Thermoregulatory behaviours and microhabitat choice can also be used to elevate body temperatures to the preferred thermal range and can lead to conserved thermal limits across environments (Watt, 1968; Chappell, 1983; Angilletta *et al*., 2002a; Buckley *et al*., 2015). Alternatively, thermal sensitivities can vary across elevation or latitudinal gradients. Although upper thermal limits tend to be highly conserved, lower thermal limits exhibit increased variance across these gradients (Sunday *et al*., 2011). Performance at lower temperatures may be particularly important for organisms at higher elevations or latitudes, where the time available for activity or development can be strongly limited (Kingsolver, 1983b; Adolph and Porter, 1993; Sinervo and Adolph, 1994).

Here, we use field experiments with *Colias* butterflies to explore how variation in morphological traits and thermal sensitivity determine patterns of flight initiation for populations along an elevation gradient. *Colias* butterflies require elevated body temperatures to initiate and maintain active flight, and use behavioural thermoregulation (including basking) to achieve these body temperatures (Watt, 1968). The time available for flight activity is limited in cooler environments, and flight time can strongly limit lifetime reproductive success for *Colias* females, especially at elevations above 2500 m (Kingsolver, 1983a; Springer and Boggs, 1986; Ellers and Boggs, 2004). For *Colias* at higher elevations, the average time available for flight activity can be <3 h per day (Kingsolver, 1983b). The importance of flight is further intensified by the short (6–10 day) adult lifespan of *Colias* butterflies in the field (Watt *et al*., 1977).

The body temperatures of *Colias* adults are strongly influenced by two morphological traits: melanin on the ventral hindwings and setal length on the ventral thorax (Watt, 1968; Kingsolver, 1983a). *Colias* populations and species at higher elevations and latitudes have increased wing melanin and setal lengths, adapting them to local climatic conditions (Watt, 1968; Roland, 1982; Kingsolver, 1983b; Ellers and Boggs, 2004). The body temperatures needed for maximal flight activity are similar for different *Colias* species and populations (34–38°C; Watt, 1968; Ellers and Boggs, 2004), but the lower thermal limits for flight initiation are less clear (Kingsolver, 1983b).

In this study, we use field experiments to quantify the relative contributions of morphology and thermal sensitivity in flight initiation for high-elevation *Colias meadii* and low-elevation *Colias eriphyle*. First, we use reciprocal transplant experiments to compare flight initiation among *Colias* species. These experiments enable us to determine how morphology, behaviour and thermal sensitivity influence body temperatures and spontaneous flight initiation across elevations. Second, we use a common garden experiment in the absence of direct solar radiation at one low elevation (1500 m) to compare differences in thermal sensitivity of flight initiation between *Colias* species. This experiment isolates physiological differences in the thermal sensitivity of flight initiation from morphological differences that may influence body temperature. By combining information from both experiments, we quantify the contributions of morphological and physiological mechanisms to local adaptation along an elevation gradient.

## Materials and methods

### Study system

*Colias* butterflies are an important model system for understanding thermal biology and local adaptation to climate because they have both morphological and behavioural mechanisms for thermoregulation. Empirical measurements and biophysical modelling confirm that darker, more melanic wings allow the butterflies to absorb more solar radiation and increase body temperatures (Watt, 1968). Likewise, longer, thicker setae on the ventral thorax can reduce convective heat loss and increase body temperatures (Kingsolver and Moffat, 1982; Kingsolver, 1983a). Although studies with tethered butterflies show that *Colias* species have similar body temperature ranges of 30–40°C for active flight (Watt, 1968), field observations of freely flying butterflies suggest that flight may be initiated at body temperatures below 30°C, especially for *Colias* species at higher elevations (Kingsolver, 1983b; Kingsolver and Watt, 1984).

We used males from two species, namely *C. eriphyle* and *C. meadii*, along an elevation gradient on the western slope of the Colorado Rocky Mountains. *Colias eriphyle* is widely distributed across western North America at a range of elevations (1400–2900 m; Springer and Boggs, 1986), whereas *C. meadii* is confined to subalpine and alpine meadows typically above 2500 m elevation in the southern Rocky Mountains (Watt, 1968). The species exhibit substantial variation in thermally important phenotypes. *Colias eriphyle* has a mean solar absorptivity of 53–60% and ventral thoracic setal length of 0.82–1.08 mm along an elevation gradient from 1700 to 2700 m (Kingsolver, 1983b). *Colias meadii* has a mean solar absorptivity of 65% and ventral thorax setae length of 1.46 mm from samples collected in central Colorado (Kingsolver, 1983b).

Our field studies involved four sites over a range of elevations in western and central Colorado, USA (Supplementary material Fig. S1). We collected *C. eriphyle* from a site near Olathe, Montrose Co., CO (N38.62, W108.02, 1600 m elevation) and another 90 km away in Gunnison, Gunnison Co., CO (N38.56, W106.94, 2300 m). We collected *C. meadii* from Cumberland Pass, Gunnison Co., CO (N38.41, W106. 29, 3600 m) and Mesa Seco in Hinsdale Co., CO (N37.59, W107.13, 3300 m–3700 m). Past studies conducted at Mesa Seco revealed differences in genotypic frequencies of the PGI locus between the lower and upper part of the mesa, a mere 500 m apart, suggesting that there may be physiological differences within this site (Watt *et al*., 2003). As a result, we distinguish individuals sampled from both below tree-line (<3300 m) and above tree-line meadows (>3400 m) within Mesa Seco.

### Micrometeorological measurements

To quantify thermal conditions and butterfly temperatures during the experiments, we measured solar radiation, wind speed and air and soil temperatures. We used a solar radiation sensor (Pace SRS-100, Moorsville, NC, USA) at plant height, an anemometer (Pace WSD-100) at 1.2 m, and thermistors (Pace PT-907) at 10 cm above the soil surface in the shade and 0.5 cm below the soil surface. An additional thermistor was modified to serve as a physical model in the sheltered environment common garden experiments (see Common garden flight initiation in the absence of direct solar radiation). The sensor was coated in epoxy, painted yellow, and paper wings were attached to mimic butterfly morphology (Kingsolver and Moffat, 1982). The epoxy models were validated using fresh butterflies with a thermocouple inserted into their thorax and recorded at 3 min intervals. The average error between the epoxy model and the butterfly was 0.6 ± 1.1°C (mean ± SD) in our test conditions, and total horizontal solar radiation was never exceeding 529 W/m^2^. Measurements were recorded every 10 s, and averaged values were output every minute using a Pace Scientific X5-SE logger.

### Reciprocal transplants: flight initiation with direct solar radiation

To quantify differences in flight initiation across the elevation gradient, we conducted reciprocal transplant experiments at a low- (Olathe) and a high-elevation site (Mesa Seco). The high-elevation site was split between a lower meadow and an upper plateau in order to explore within-site variability. Twelve *C. meadii* from Mesa Seco were brought to Olathe (1600 m), and 12 *C. eriphyle* from Olathe were brought to Mesa Seco (from 3300 and 3600 m), where they were compared with the local populations. At each site, 24 open-bottomed cages were placed on top of vegetation and in areas shielded from direct wind. The cages were cylinders 30 cm in diameter and 60 cm tall, constructed of SeeVue (Phifer^®^, Tuscaloosa, AL, USA) window screen topped with bridal veil and positioned using garden staples. The screen reduced solar radiation by <15%. A single animal was placed on the vegetation at the bottom of each enclosure prior to local sunrise. Cages were checked every 2 min, and the time of spontaneous flight initiation was recorded. The measure of spontaneous flight initiation indicated not only when the butterflies were capable of flight but also when they were willing to begin flying. This motivation (or lack of it) captured the behavioural aspect of flight initiation. At Mesa Seco, a portable weather station (see Micrometeorological measurements) was placed at the middle of the site to record air and soil temperatures, solar radiation and wind speed. The experiments with *C. meadii* and *C. eriphyle* were repeated twice at each site on different days in July 2011. We also compared populations within Mesa Seco, transplanting six individuals collected below the tree line and six collected above the tree line to the experimental at both 3300 and 3600 m. These experiments were repeated five times on different days in July and August 2011.

The environmental data collected during each of the transplant experiments were combined with measures of the thermally important traits (absorptivity of the ventral hindwings and thoracic fur thickness) to predict the body temperature at the time of flight initiation for each individual. We used an established and validated biophysical model for *Colias* to predict steady-state body temperatures (also see Kingsolver and Moffat, 1982; Kingsolver, 1983b; Tsuji *et al*., 1986; Buckley and Kingsolver, 2012). We used a steady-state (rather than transient) model because the thermal response time (time constant) for *Colias* is typically <60 s (Kingsolver, 1983a). Environmental parameters were averaged over 6 min prior to flight initiation. Details of the model are provided by Buckley and Kingsolver (2012). Biophysical models predict that for a basking butterfly, body temperature should be directly proportional to air temperature and to the direct solar radiative heat flux density (Kingsolver and Moffat, 1982; Kingsolver, 1983b). Thus, we used our biophysical model and micrometeorological data to quantify the relationship of predicted basking temperature to air temperature and direct solar radiation.

### Common garden flight initiation in the absence of direct solar radiation

Differences in both morphological traits and thermal sensitivity among populations and species may contribute to differences in the timing of flight initiation during the reciprocal transplant experiments. To isolate differences in thermal sensitivity, we used a common garden experiment with closed tents that blocked direct solar radiation, largely eliminating the effects of wing melanin and setal length on body temperature. All trials were conducted at a lower-elevation site in Montrose (N38.46, W107.88, 1500 m) to ensure that temperatures in the tent were high enough to elicit flight. We performed two sets of common garden experiments: one comparing populations of *C. eriphyle* from Olathe (1600 m) and Gunnison (2300 m); and the other comparing *C. eriphyle* from 1600 m (Olathe) and *C. meadii* from 3300–3600 m (Mesa Seco and Cumberland Pass). Each trial was conducted in three 2.75 m × 3.35 m nylon enclosures (Kelty^®^ medium portable shelters, Boulder, CO, USA) that reduced solar radiation by 65% on average. The tent also greatly reduced wind and wind gusts experienced by the butterflies. A portable weather station (see Micrometeorological measurements) was placed inside the middle tent and set to record air and soil temperatures, solar radiation and wind speed at 1 min intervals.

Body temperature at spontaneous flight initiation was estimated by recording the temperature of the physical butterfly model (see above) at 1 min intervals. We used the physical model temperatures (rather than estimates of body temperature from a mathematical model) because they accurately indicate temperatures in the tents, which blocked direct solar radiation and thus minimized temperature differences associated with behaviour and solar absorption. Each set of experiments was repeated five times on different days in July and August 2012. Individuals were held at ~3°C until the start of the assay. They were then placed in the centre of the tent before local sunrise, with a researcher in the southwest corner. If an individual initiated flight, the decimal hour was recorded. It was also noted if an individual did not initiate flight over the course of the trial.

### Statistical analyses

All statistical analyses were conducted in R (version 2.15.2) (R Core Team, 2014). For the reciprocal transplant experiments, the probability of flight initiation (for all individuals) and the time of flight initiation (for those individuals that initiated flight) were analysed in a linear mixed model framework, nlme (Pinheiro *et al*., 2014), with population (or species) and elevation as main effects and Julian date of the trial nested within site as a random intercept. The predicted basking temperature of flight initiation was analysed with population (or species) and elevation as fixed effects and with Julian date of the trial as a random intercept.

For the common garden experiments, the probability of flight initiation was modelled as a binomial response (flight or no flight) using a generalized linear model. Given that environmental conditions determine the rate of heating in the tent, we included the initial morning temperature (mean air temperature inside the tent from 07.30 to 07.45 mountain daylight time) and population (or species) as fixed effects. Tent was nested within Julian date as a random effect to account for between-tent and between-day variance. Significance testing was performed by comparing simpler models (a single predictor variable or both predictor variables without an interaction) with the full model (including an interaction term) using χ^2^ tests. Time of flight initiation and temperature of flight initiation were analysed using linear mixed-effects models with the same fixed and random effects and tested using ANOVAs.

## Results

### Reciprocal transplants: flight initiation with solar radiation

**Figure 1:**
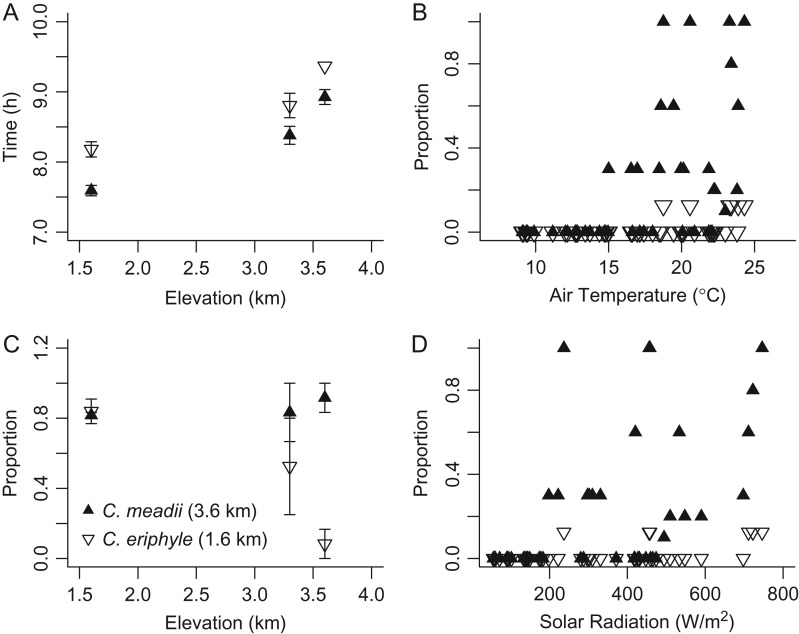
Reciprocal transplants between *Colias meadii* and *Colias eriphyle* show the proportion of butterflies that initiated flight (mean ± SEM; **A**) and the time at initiation (hour, mean ± SEM; **C**) for each population as a function of the elevation (in metres) of the observation site. To show how air temperature and direct solar radiation determine these proportions, we selected a representative day (26 July 2011) at Mesa Seco (3.6 km) and show the proportion initiated at a given air temperature (**B**) and level of direct solar radiation (**D**).

In the reciprocal transplant, the proportion of *C. eriphyle* that initiated flight decreased significantly with increasing elevation, and the proportion of *C. meadii* that initiated flight remained constant across elevations, producing a significant interactive effect (species, χ^2^_(2, *n*=83)_ = 17.39, *P* < 0.001; trial elevation, χ^2^_(2, *n*=83)_ = 11.83, *P* = 0.02; interaction, χ^2^_(1, *n*=83)_ = 17.39, *P* = 0.005; Fig. 1C). Moreover, the *C. meadii* initiated flight significantly earlier than *C. eriphyle* (*F*_(1,54)_ = 7.99, *P* < 0.05) regardless of trial elevation (*F*_(1,54)_ = 42.57, *P* = 0.09; Fig. 1A). This confirms that high-elevation *C. meadii* are more likely to initiate flight and to fly earlier than low-elevation *C. eriphyle* across ambient temperatures and elevations. The direct influences of ambient temperatures and solar radiation are shown for a representative day at the high-elevation site (Fig. 1B and D).

When we compared *C. meadii* collected from below and above the tree line at Mesa Seco (3300 and 3600 m), we saw no significant difference in the proportion of butterflies that initiated flight (collection site, χ^2^_(2, *n*=116)_ = 3.91, *P* = 0.14; trial elevation, χ^2^_(2, *n*=116)_ = 4.35, *P* = 0.11; interaction, χ^2^_(1, *n*=116)_ = 1.09, *P* = 0.29; Supplementary material Fig. S2A) and no effect of collection site (*F*_(1,82)_ = 0.15, *P* = 0.87; Supplementary material Fig. S2A). The time of initiation was significantly later at the higher trial elevation regardless of collection site (*F*_(1,82)_ = 4.46, *P* = 0.03 for trial elevation; *F*_(1,82)_ = 0.03, *P* = 0.85 for the interaction; Supplementary material Fig. S2B).

**Figure 2:**
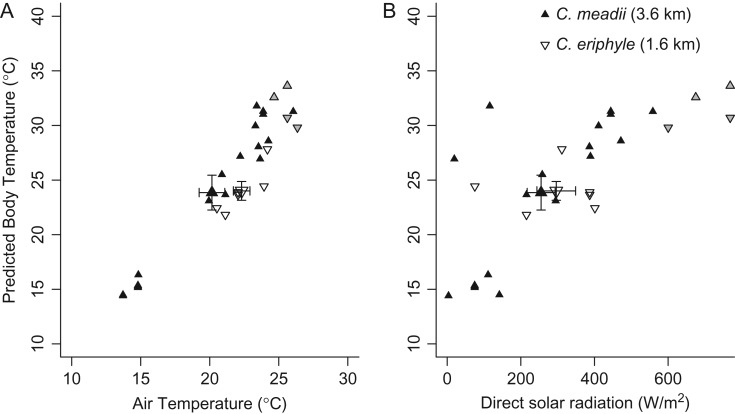
The predicted basking temperatures at flight initiation, taking into account that morphological differences for *C. eriphyle* (open symbols) and *C. meadii* (filled symbols) at Mesa Seco do not differ significantly between species on all days. The larger symbol depicts the mean ± SEM for both the air temperature (**A**) and solar radiation (**B**) and the predicted basking body temperature of individuals of each species.

We estimated the body temperature at flight initiation for the high-elevation trials using the average phenotype for the *C. meadii* (mean ± SD = 69.1 ± 3.3% absorptivity and 1.27 ± 0.23 mm thoracic setal length) and the average phenotype for *C. eriphyle* (51.8 ± 7.0% absorptivity and 0.63 ± 0.18 mm thoracic setal length). After restricting our analysis to animals that initiated flight, it included 20 *C. meadii* and six *C. eriphyle*. As expected, the predicted basking temperature increased linearly with increasing air temperature and increasing direct solar radiation (Fig. 2). The absence of data points for *C. eriphyle* at low air temperatures reflects the fact *C. eriphyle* fail to achieve the body temperatures needed to initiate flight at low air temperatures (compare Figs 1 and 2). Of the butterflies that were able to initiate flight, there was no species difference in the body temperature at initiation (*F*_(1,23)_ = 0.22, *P* = 0.64; Fig. 2). Butterflies initiated flight at cooler air temperatures at the highest elevation (*F*_(1,23)_ = 6.48, *P* = 0.02), but there was no interaction between species and elevation (*F*_(1,23)_ = 1.38, *P* = 0.25).

It was also useful to consider the distributions of predicted basking temperatures for both fliers and non-fliers of each species in the reciprocal transplant experiments at Mesa Seco on the days for which we have all relevant data (Supplementary material Fig. S3). In the common environmental conditions of these experiments, *C. eriphyle* fly much less frequently than *C. meadii* because *C. eriphyle* are rarely able to achieve the body temperatures needed for flight (Supplementary material Fig. S3).

### Common garden flight initiation in the absence of direct solar radiation

The common garden experiments allowed us to look at flight initiation in a controlled environment in the absence of direct solar radiation. Populations of *C. eriphyle* from 1600 m (Olathe) and 2300 m (Gunnison) showed little difference in the thermal sensitivity of flight initiation (Supplementary material Fig. S4). *Colias eriphyle* from Gunnison were significantly more likely to initiate active flight (χ^2^_(2, *n*=178)_ = 9.05, *P* = 0.01) independent of the initial air temperature (χ^2^_(2, *n*=178)_ = 3.7, *P* =  0.15; Supplementary material Fig. S4A) measured at the start of the trial. Of those that did initiate flight, there was no significant difference in the timing of flight initiation as a function of population (*F*_(1,138)_ = 1.41, *P* = 0.23), initial air temperature (*F*_(1,138)_ = 0.16, *P* = 0.71) or the interaction (*F*_(1,138)_ = 0.47, *P* = 0.49; Supplementary material Fig. S4B). The butterfly model temperature at flight initiation showed no significant effect of population (*F*_(1,138)_ = 2.57, *P* = 0.11; Supplementary material Fig. S4D) nor was there an interaction between population and initial air temperature (*F*_(1,138)_ = 0.88, *P* = 0.34; Supplementary material Fig. S4C).

**Figure 3:**
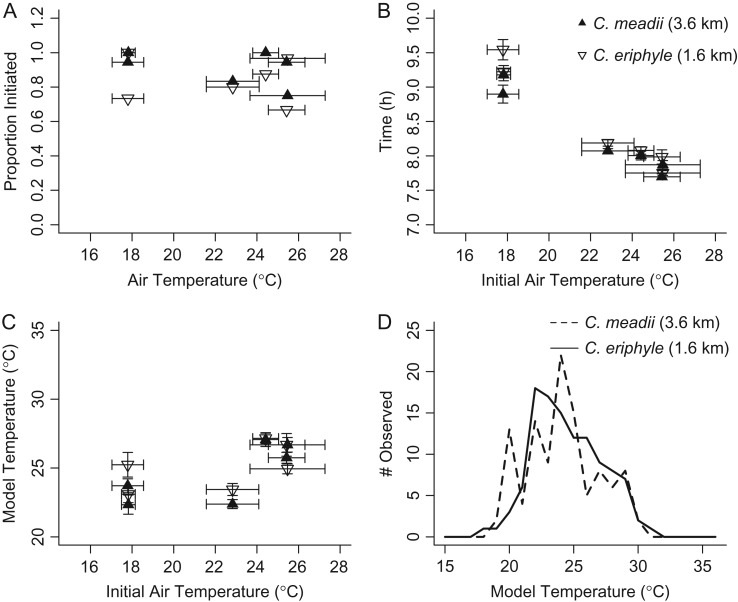
Results from the common garden between *C. eriphyle* (open symbols) and *C. meadii* (closed symbols). (**A**) The two species do not differ significantly in their probability of flight initiation (mean ± 95% confidence intervals). (**B**) Cooler initial temperatures lead to later flight initiation times (hour, means ± SEM) in both species. (**C**) *Colias meadii* initiate flight at cooler temperatures (in degrees Celsius, means ± SEM). (**D**) The distributions of initiation temperatures binned by 0.5°C for the two species.

When we compare *C. eriphyle* from 1600 m and *C. meadii* from 3500 and 3600 m in the absence of direct solar radiation, we see that *C. meadii* initiated flight at slightly cooler temperatures relative to the low-elevation *C. eriphyle*. We detected no significant difference in the probability of flight initiation (Fig. 3A; χ^2^_(2, *n*=249)_ = 4.06, *P* = 0.13 for species; χ^2^_(2, *n*=249)_ = 3.01, *P* = 0.22 for collection site; χ^2^_(1, *n*=249)_ = 1.95, *P* = 0.16 for the interaction). *Colias meadii* initiated flight significantly earlier than *C. eriphyle* (Fig. 3B; *F*_(1,220)_ = 4.21, *P* = 0.04); this effect was most clearly seen on cool mornings. All butterflies initiated flight later on cooler mornings (Fig. 3C; *F*_(1,220)_ = 373.64, *P* < 0.05). *Colias meadii* also initiated flight at significantly lower model temperatures than *C. eriphyle* (Fig. 3D; *F*_(1,220)_ = 4.64, *P* = 0.03) regardless of the initial morning air temperatures (*F*_(1,220)_ = 5.54, *P* = 0.07), and there was no interaction between species and initial morning temperature (*F*_(1,220)_ = 1.95, *P* = 0.16). This suggests that *C. meadii* initiates flight earlier and at lower body temperatures than *C. eriphyle*, and both species frequently initiated flight at body temperatures of 20–25°C.

## Discussion

### Thermal limits on activity and fitness

In *Colias* and many other ectotherms, populations and species are locally adapted to enable activity in different environmental conditions. Previous work along an elevation gradient showed that *C. eriphyle* from higher elevations are able to initiate flight earlier than those from lower elevations, owing to differences in wing melanin among populations (Ellers and Boggs, 2004). These effects are even more striking when differences between species are considered. For example, our reciprocal transplants with *C. eriphyle* and *C. meadii* at high elevation (3600 m) reveal that <10% of *C. eriphyle* are able to initiate flight at all in these cool environmental conditions, in contrast to resident *C. meadii* (91% flight initiation). Failure to fly not only reduces potential mating and reproductive success, but can have immediate fitness consequences; individuals in our field experiments that were not able to achieve flight were often subject to predation by ants and wasps (H. J. MacLean, personal observation; Roland, 2006). For montane and alpine *Colias*, low environmental temperatures put a premium on achieving flight at cooler temperatures to maximize the time available for activity. As expected, the body temperature of basking butterflies increases with both increasing air temperature and increasing direct solar radiation (Fig. 2). This finding aligns with observations for other high-altitude *Colias* species (Roland, 1982). In both experiments, *C. meadii* initiated flight at cooler air temperatures and a higher proportion at high-elevation sites relative to low-elevation sites, demonstrating local adaptation to their high-elevation environment. These results affirm the importance of local adaptation for flight activity and fitness in this system. As discussed below, our experiments demonstrate that both physiological and morphological differences among species contribute to this local adaptation.

### Physiological determinants of performance

Numerous studies have documented physiological differences in thermal performance curves for ectotherms from different climatic regions and thermal environments. However, most of these data and patterns are based on laboratory measurements of performance (Frazier *et al*., 2006; Deutsch *et al*., 2008; Angilletta, 2009; Sunday *et al*., 2011). Using these laboratory estimates to predict performance and fitness in field conditions can be problematic, especially for lower and upper thermal limits (Kearney *et al*., 2009b; Kingsolver *et al*., 2013). Additionally, studies predicting the activity duration for ectotherms across large geographical scales often use data on thermal tolerance to predict thermal performance breadth (Adolph and Porter, 1993; Kearney and Porter, 2009; Buckley and Kingsolver, 2012), adding uncertainty about predictions for lower and upper thermal limits for performance (Kingsolver *et al*., 2013). To avoid these issues, we used field experiments to compare the lower thermal limits for flight initiation between *Colias* populations and species.

Previous studies with *Colias* using tethered butterflies in the field show similar thermal optima for flight across species (34–38°C) and that flight rarely occurs at body temperatures below 28–30°C (Watt, 1969). Observations of free-flying *Colias* indicate that flight activity increases when basking body temperatures are above 30°C, but there is some flight activity for *C. eriphyle* and *C. meadii* at basking temperatures of 28–30°C (Kingsolver, 1983a). Our common garden experiments yielded two important results about the thermal biology of *Colias*. First, the average body temperature for flight initiation for *Colias* was between 24–26°C in our experiments, 4°C lower than observed in previous studies. This highlights the importance of measuring temperatures for initiating activity in addition to thermal optima. Second, and in contrast to previous studies, our results also indicate a small but significant difference in the thermal sensitivity of flight initiation between high-elevation *C. meadii* and lower-elevation *C. meadii*. Our common garden experiments (in the absence of direct solar radiation) show that *C. meadii* initiate flight at lower body temperatures (0.8°C on average) than *C. eriphyle*. High-elevation species occupy cool environments and have reduced lower thermal limits for flight initiation, which increases the time available for flight. The differences in lower thermal limits for flight initiation between *C. eriphyle* and *C. meadii* that we report here may seem modest, but recent biophysical and demographic modelling in this system shows that the assumption of lower thermal limits for flight can have major effects on predictions of activity time, reproduction and fitness at high-elevation sites (Buckley and Kingsolver, 2012). For example, using the previous information on thermal limits for *Colias*, these models predicted that *C. meadii* at alpine sites in Colorado (elevation 3500 m) would be unable to maintain populations (i.e. the predicted mean fitness was below the replacement rate; Buckley and Kingsolver, 2012). Incorporating the values for lower thermal limits reported here into these models would increase the predicted mean fitness reported for *C. meadii* at these high-elevation sites.

We note that, as with many aspects of performance, distinguishing behavioural from physiological components of thermal sensitivity of flight initiation is difficult or impossible here. For example, *C. meadii* may be more strongly motivated to fly in marginal environmental conditions (hence, at lower body temperatures) than *C. eriphyle*. Given the greater restrictions on available flight time for *C. meadii* than *C. eriphyle* in their respective habitats (Kingsolver, 1983a), one might expect *C. meadii* to possess high levels of behavioural motivation for flight whenever possible. More mechanistic studies would be needed to understand the interplay of behaviour and physiology in determining these differences in thermal sensitivity of flight.

Several factors may contribute to the differences between our present results and those from previous studies. Watt (1968) measured body temperatures of tethered butterflies implanted with thermistors repeatedly during sunny conditions in the field to determine the percentage of time in flight as a function of body temperature. Tethering may reduce the frequency of flight (Kutsch and Stevenson, 1981), as animals discover that flight does not lead to sustained movement. For example, even at body temperatures near the optimum (34–38°C), the frequency of flight was only 25–33% in *C. eriphyle* and 15–33% in *C. meadii* (Watt, 1968). In contrast, studies of freely flying *C. eriphyle* suggested that when body temperature exceeds 30°C, males spend >90% of their available time in flight (Kingsolver, 1983a). If tethering reduces the frequency of flight, especially in non-optimal thermal conditions, this could potentially bias estimates of lower limits on flight activity. We observed that once an individual first initiated flight, he would fly several additional times and then remain perched on the side of the cage or tent, suggesting that the motivation for flight is reduced in these spatially confining situations. To account for the potential lack of motivation for flight in a confined space, we focused on the time and temperature of initial flight, rather than the mean frequency of flight during the trial.

Another reason why our results may differ is that we estimated or predicted basking temperatures during basking, whereas Watt (1968) measured body temperatures during flight. Flight temperatures may exceed basking temperatures, especially in conditions of high solar radiation for tethered butterflies. Some individuals of *C. eriphyle* and *C. meadii* had flight temperatures of 42–44°C (Watt, 1968), well above the basking temperatures typically seen for *Colias* at these elevations (Kingsolver, 1983b). Conversely, Kingsolver (1983a, b) assayed patterns of flight activity for freely flying butterflies by repeatedly counting the number of individuals crossing a transect line at different times during the day and relating this to the measured and predicted basking temperatures. This assay would not detect short, initial flights of butterflies in marginal conditions, and thus will overestimate the minimal body temperature needed for flight initiation.

### Morphological and physiological contributions to local adaptation

Although many studies have documented local adaptation to climate via differences in the thermal sensitivity of performance (Hertz *et al*., 1983; Stevenson, 1985; Navas, 1996; Angilletta *et al*., 2002b) or morphological differences (Berry and Willmer, 1986; Ellers and Boggs, 2004), few studies have explored these two mechanisms simultaneously (Frazier *et al*., 2008). To our knowledge, no studies have attempted to quantify their relative contributions.

Our reciprocal transplant experiments, performed in the presence of direction solar radiation, allowed us to distinguish the contributions of morphological and physiological mechanisms of local climatic adaptation of *Colias* species along the elevation gradient. The effects on flight initiation are greatest for transplants between the lighter low-elevation species and the darker high-elevation species (as seen in Fig. 1). For example, at the highest elevation site (3600 m), >90% of the resident *C. meadii* are able to initiate flight, whereas <10% of low-elevation (1600 m) *C. eriphyle* ever achieved flight in these conditions (Fig. 1C). Moreover, the high-elevation individuals initiated flight at a higher proportion at the highest elevation site, relative to their performance at the lower-elevation sites, creating an interaction. For individuals that did initiate flight, initiation occurred earlier (on average 35 min) for *C. meadii* than for *C. eriphyle* at all sites (Fig. 1D). These results confirm the findings of previous work in documenting the importance of morphological differences in wing melanin and thoracic insulation for local adaptation in *Colias* (Watt, 1968; Kingsolver, 1983b; Ellers and Boggs, 2004).

By combining the results of our common garden and reciprocal transplant experiments, we can quantify the relative contributions of these two mechanisms to differences in flight initiation at the low-elevation site. Differences in flight initiation reflect both physiological and morphological differences in the reciprocal transplants (direct solar radiation present), but only physiological differences in thermal sensitivity in the common garden experiment (direct solar radiation absent). At the low-elevation site, the higher elevation species initiates flight 5 min earlier without direct solar radiation and 35 min earlier with direct solar radiation. Thus, differences in both thermal sensitivity (15%) and morphology (85%) contributed to the differences in flight performance between the two species in the field. The morphological differences that provide high-elevation butterflies with darker wings, thicker thorax setae and, ultimately, higher body temperatures help butterflies to achieve flight sooner and, ultimately, greater fitness. However, because of environmental variability and the short window during which these animals are adults, it appears that adaptation also occurs, to a lesser degree, at a physiological level. Thus, both morphology and thermal sensitivity contribute to local adaptation.

Consideration of the contributions of local adaptation in morphology, physiology and behaviour may be crucial for accurate forecasting of responses to future climates. The type and strength of selection imposed by rapid climate change is likely to vary among populations (Hoffmann and Sgrò, 2011). Populations can respond to climate change by shifting their distribution, evolving higher thermal tolerance or adapting to greater environmental variability, and it is likely that successful populations will use a combination of these tactics (Bradshaw and Holzapfel, 2006; Parmesan, 2006). Moreover, climatic change is not limited to the growing season. Warming winters may pose a significant challenge for other life stages (Radchuk *et al*., 2013; Williams *et al*., 2015). Differences in the strategies by which populations and species respond may account for the individualistic abundance, phenology and distribution shifts observed in response to recent climate change (Williams *et al*., 2007). Our findings suggest the importance of measuring lower limits in addition to the optima of performance curves.

## Supplementary material

Supplementary material is available at *Conservation Physiology* online.

## Supplementary Material

Supplementary Data
