# Supplementary material for: Morphological and physiological determinants of local adaptation to climate in Rocky Mountain butterflies
Source: Conserv Physiol. 2016 Sep 22;4(1):cow035. doi: 10.1093/conphys/cow035 (PMC5033134; doi:10.1093/conphys/cow035)
Supplement: Supplementary Data [file cow035_conphys-2015-072.pdf]

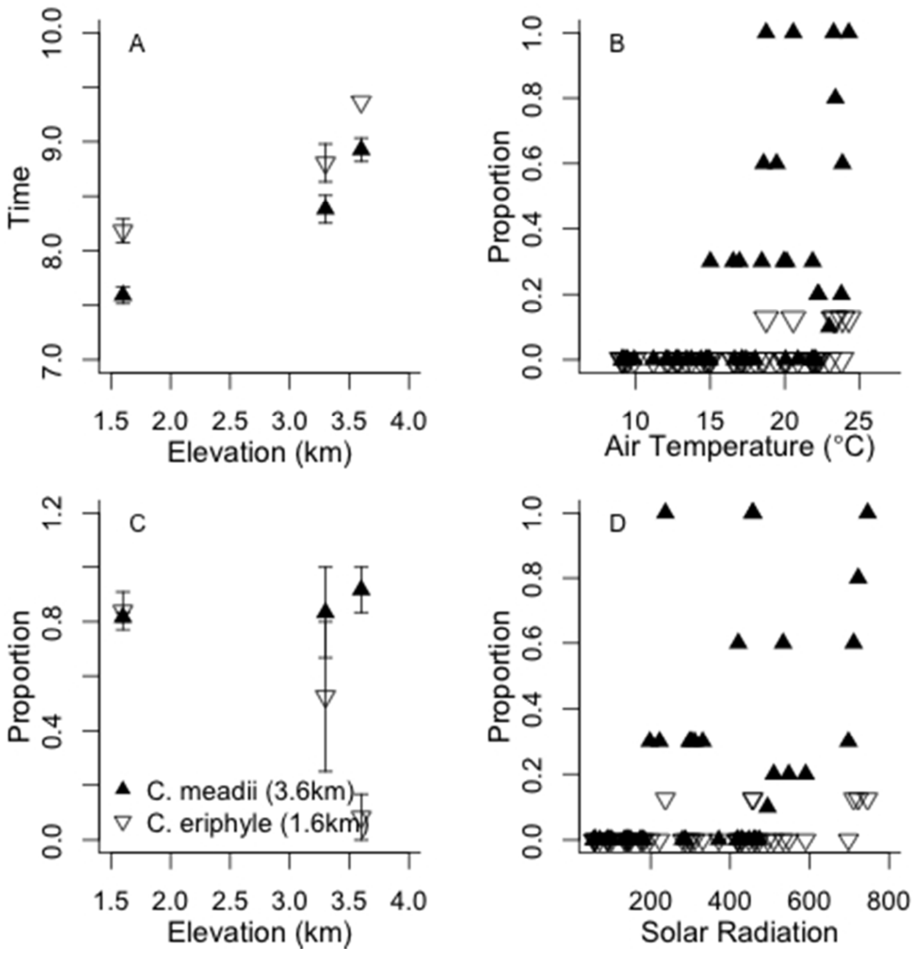

Reciprocal transplants between C.meadii and C. eriphyle: A) show the proportion of butterflies that initiated (mean+se) and C) the time at initiation (hour, mean +se) for each population as a function of the elevation (m) of the observation site. To show how air temperature and solar radiation determine these proportions, we selected a representative day (July 26, 2011) and show the proportion initiated at given B) air temperature and D) level of solar radiation

Fig. 1

169x169mm (72 x 72 DPI)

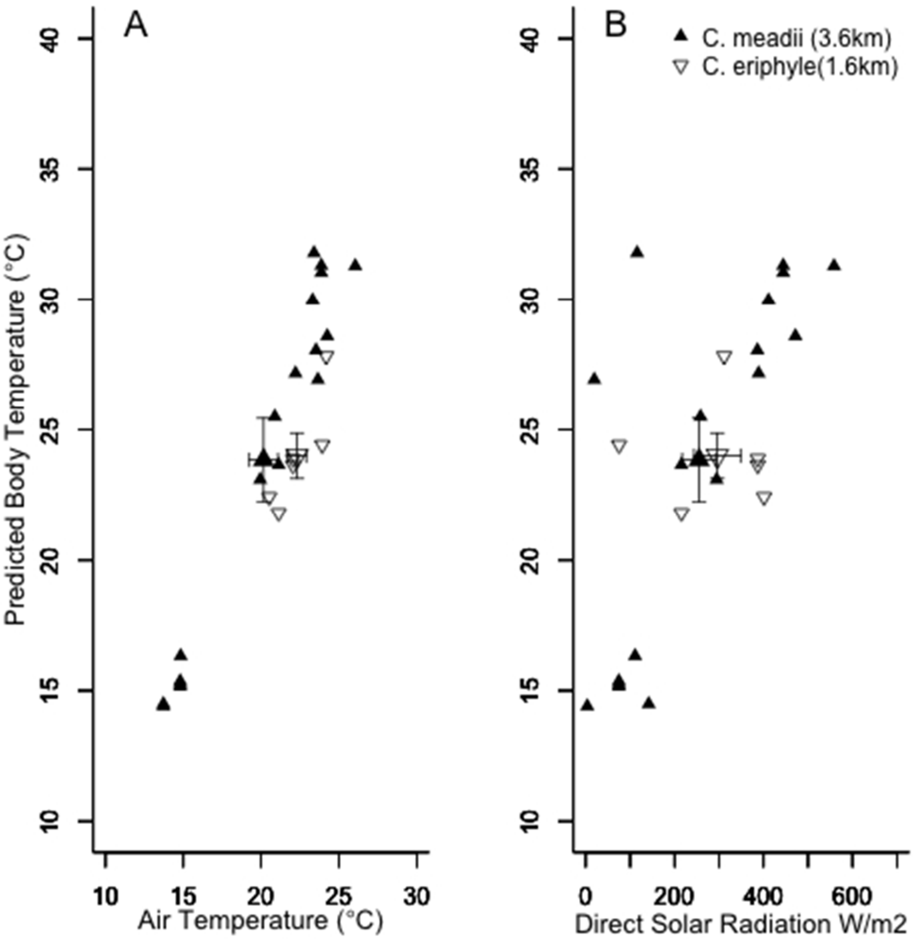

Reciprocal transplants between C.meadii and C. eriphyle: A) show the proportion of butterflies that initiated (mean+se) and C) the time at initiation (hour, mean +se) for each population as a function of the elevation (m) of the observation site. To show how air temperature and solar radiation determine these proportions, we selected a representative day (July 26, 2011) and show the proportion initiated at given B) air temperature and D) level of solar radiation

Fig. 2

169x169mm (72 x 72 DPI)

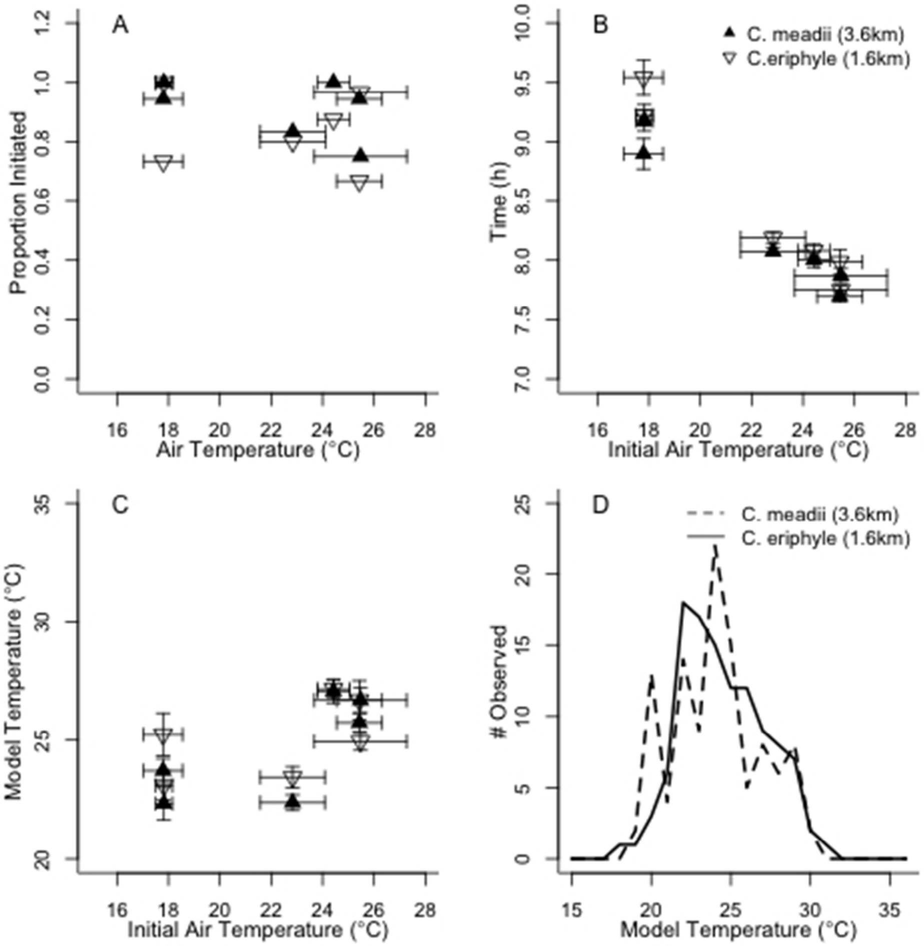

Results from the common garden between C. eriphyle (open symbols) and C. meadii (closed symbols). a) The two species do not differ significantly in their probability of flight initiation (mean + 95% confidence intervals). b) Cooler initial temperatures lead to later flight initiation times (hour, means+se) in both species. c) C. meadii initiate flight at cooler temperatures (°C, means + se). d) The distributions of initiation temperatures binned by 0.5°C for the two species.

Fig. 3

169x169mm (72 x 72 DPI)

Morphological and physiological determinants of local adaptation to climate in Rocky Mountain butterflies

Supplementary Materials:

SFigure 1: Map of all four collection sites showing name and elevation. *Colias eriphyle* were collected from Olathe and Gunnison whereas *C. meadii* were collected from Cumberland Pass and Mesa Seco.

SFigure 2: Results from the reciprocal transplant between *C. meadii* collected above and below tree-line within Mesa Seco. A) the proportion initiated (mean $\pm$ se) and B) the time at initiation (hour, mean $\pm$ se) for each population as a function of the elevation (m) of the observation site.

SFigure 3: Results from the common garden between two populations of *C. eriphyle*. A) The higher elevation population exhibits a higher probability of initiation (mean $\pm$ 95% confidence intervals) B) Both populations initiate at a similar time (hour, means $\pm$ se). C) Body temperatures at initiation (means $\pm$ se) are warmer when air temperatures are warmer. The flight metrics are plotted as a function of air temperature (means $\pm$ se). D) The distributions of initiation temperatures are binned by 0.5˚C for the two populations.

SFigure 4: Results from the reciprocal transplant between *C. meadii* and *C. eriphyle* at Mesa Seco. Each panel corresponds to each species. The light bars are the predicted body temperatures for basking butterflies over the course of the morning (at 3 minute intervals) and the dark bars indicate the number of butterflies who initiated flight at those temperatures.

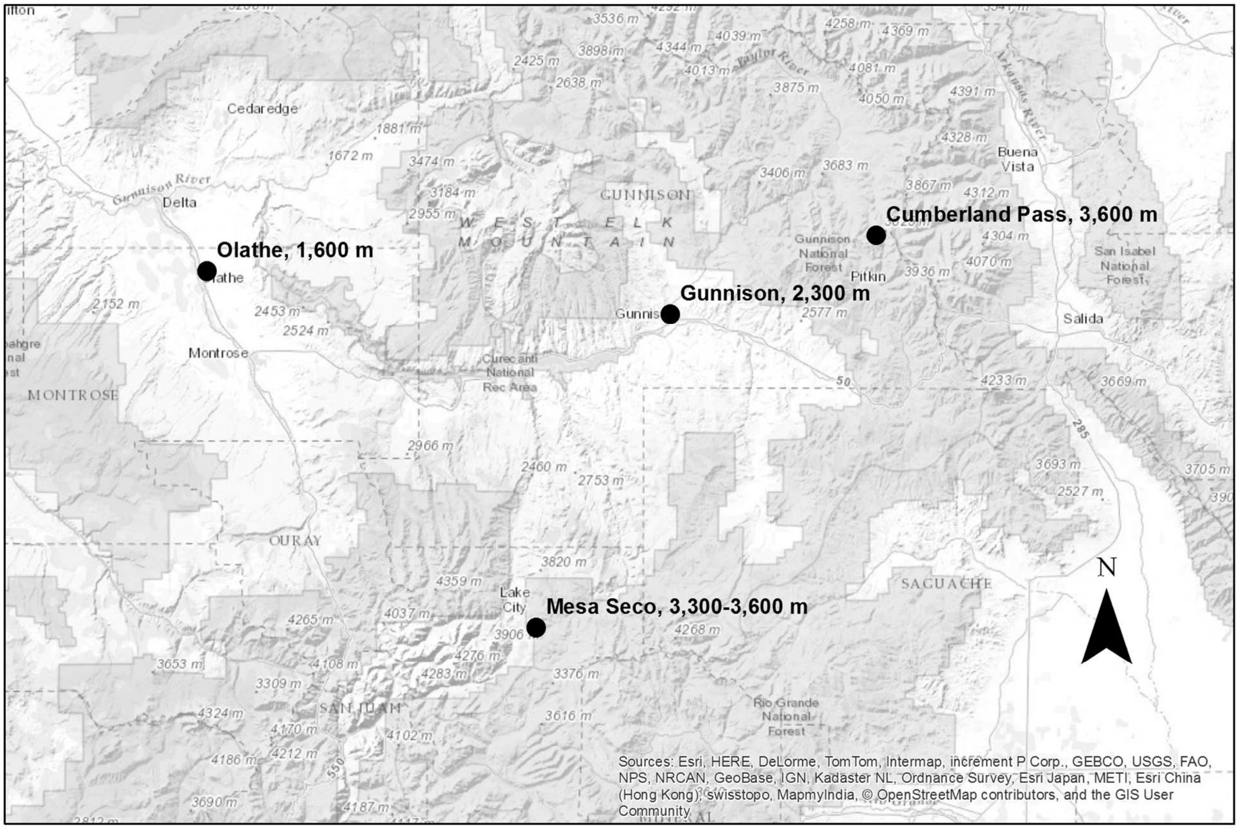
Olathe, 1,600 m
Gunnison, 2,300 m
Cumberland Pass, 3,600 m
Mesa Seco, 3,300-3,600 m
N
Cedaredge
Delta
Gunnison River
Montrose
MONTROSE
OURAY
GUNNISON
W E S T E L K
M O U N T A I N
Curecanti National Rec Area
Gunnison National Forest
Pitkin
Buena Vista
San Isabel National Forest
Salida
Lake City
SAGUACHE
SAN JUAN
Rio Grande National Forest
Taylor River
Arkansas River
Sources: Esri, HERE, DeLorme, TomTom, Intermap, increment P Corp., GEBCO, USGS, FAO, NPS, NRCAN, GeoBase, IGN, Kadaster NL, Ordnance Survey, Esri Japan, METI, Esri China (Hong Kong), swisstopo, MapmyIndia, © OpenStreetMap contributors, and the GIS User Community

SFigure 1:

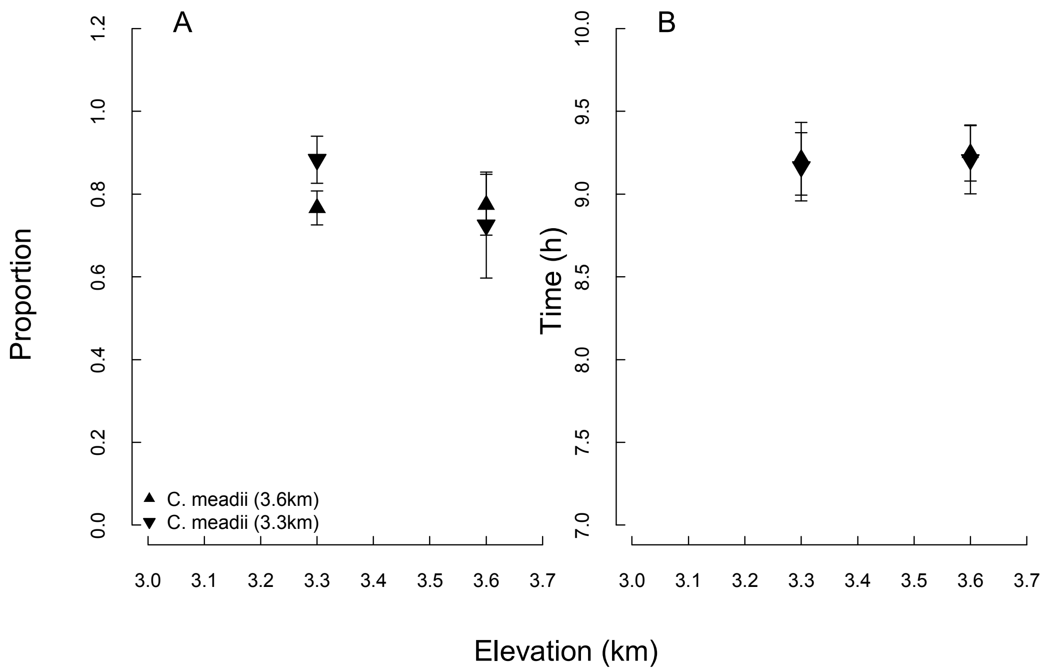
A
B
Proportion
Time (h)
C. meadii (3.6km)
C. meadii (3.3km)
Elevation (km)

SFigure 2:

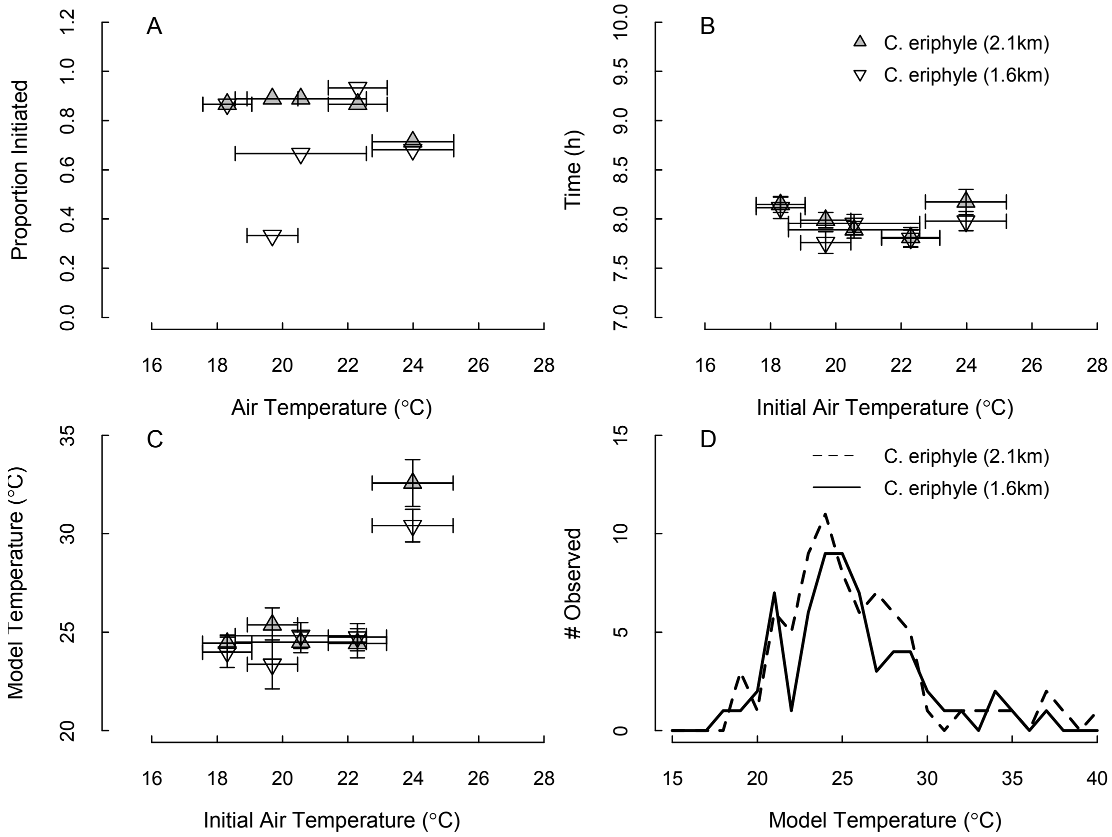

SFigure 3

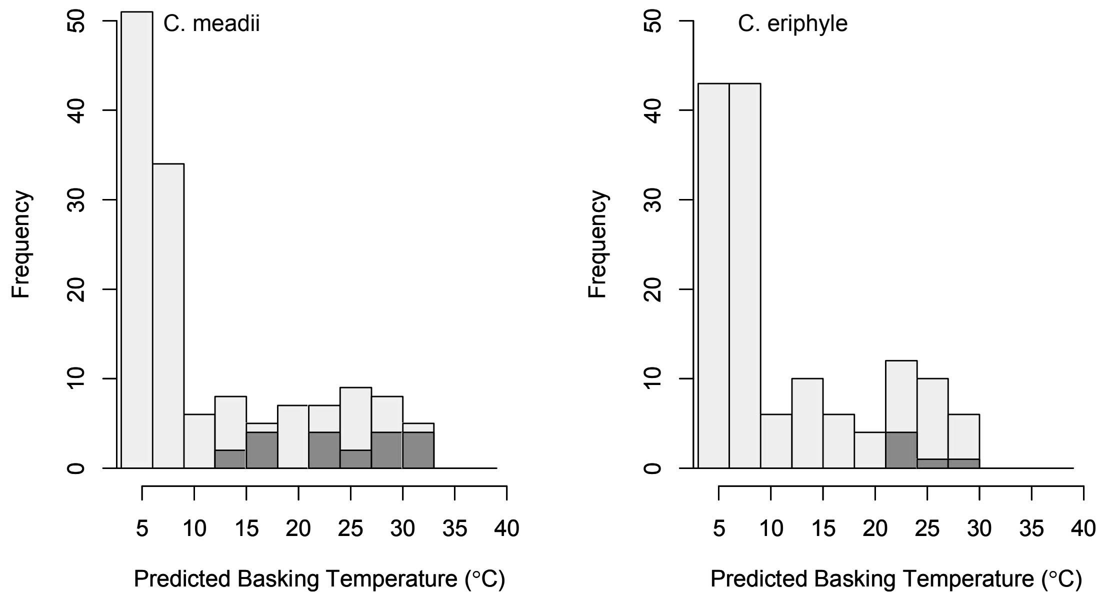

SFigure 4
